# Geminivirus–Host Interactions: Action and Reaction in Receptor-Mediated Antiviral Immunity

**DOI:** 10.3390/v13050840

**Published:** 2021-05-06

**Authors:** Marco Aurélio Ferreira, Ruan M. Teixeira, Elizabeth P. B. Fontes

**Affiliations:** Department of Biochemistry and Molecular Biology, BIOAGRO, National Institute of Science and Technology in Plant–Pest Interactions, Universidade Federal de Viçosa, Viçosa 36571.000, MG, Brazil; marco.aurelioferreira@hotmail.com (M.A.F.); ruanmaloni@gmail.com (R.M.T.)

**Keywords:** PAMP-triggered immunity, effector-triggered immunity, NIK1 antiviral defense, viral suppressors, effectors, NIK1, PTI, ETI, geminiviruses

## Abstract

In plant−virus interactions, the plant immune system and virulence strategies are under constant pressure for dominance, and the balance of these opposing selection pressures can result in disease or resistance. The naturally evolving plant antiviral immune defense consists of a multilayered perception system represented by pattern recognition receptors (PRR) and resistance (R) proteins similarly to the nonviral pathogen innate defenses. Another layer of antiviral immunity, signaling via a cell surface receptor-like kinase to inhibit host and viral mRNA translation, has been identified as a virulence target of the geminivirus nuclear shuttle protein. The *Geminiviridae* family comprises broad-host range viruses that cause devastating plant diseases in a large variety of relevant crops and vegetables and hence have evolved a repertoire of immune-suppressing functions. In this review, we discuss the primary layers of the receptor-mediated antiviral immune system, focusing on the mechanisms developed by geminiviruses to overcome plant immunity.

## 1. Introduction

Plants, like any other organism, are frequently exposed to infections caused by a diversity of pathogens. The microbe perception by the plant via cell surface and intracellular receptors is crucial for the activation of plant defenses during pathogen attack [1,2]. Conversely, the recognition of the host enables the pathogen to activate virulence strategies. The receptor-mediated innate immune system is broadly divided into two lines of defense. The first line of plant defense against pathogens is represented by cell surface-localized pattern recognition receptors (PRRs), which sense and recognize pathogen-associated molecular patterns (PAMPs) presented by the pathogens, or danger-associated molecular patterns (DAMPs), endogenous signals released by the host upon infection [3,4]. Upon PAMP recognition, PRRs are activated to initiate PAMP-triggered immunity (PTI), a relatively weak defense barrier that inhibits most invading organisms [5,6]. Recent studies have demonstrated that viruses, like nonviral pathogens, both activate and suppress PTI-like responses [7]. Therefore, successful infection depends on PTI suppression by virulence effectors eliciting effector-triggered susceptibility (ETS) [8,9,10].

To overcome these virulence strategies, plant cells have evolved a second line of defense called effector-triggered immunity (ETI), activated in resistant genotypes upon specific interactions between host intracellular receptors (resistance proteins; R) and pathogen avirulence (Avr) effectors [11]. Therefore, many effectors that activate ETI (avirulence factors) in resistant genotypes have evolved to suppress PTI as virulence factors [8,12]. The nonviral pathogens effectors are delivered into the apoplast or injected into the cytoplasm of plant cells via a microbial secretion system [13]. In contrast, viral effectors are synthesized intracellularly and promote virulence by interfering with the components of the host defense system [7,14]. Due to the limited coding capacity of viral genomes, virtually all virus-encoded proteins, including essential proteins required for infection, can function as avirulence factors in genotypes harboring the cognate R gene. Therefore, viral avirulence factors are often required for a successful infection and almost invariably act as virulence factors in susceptible hosts. Viral effectors are considered here as virus-encoded proteins that interfere with host defenses to promote virulence.

Another layer of the antiviral immunity corresponds to the transmembrane receptor-like kinase nuclear shuttle protein (NSP)-interacting kinase 1 (NIK1)-mediated antiviral defense that is often suppressed by the geminivirus nuclear shuttle protein (NSP) [15]. The NIK1-mediated antiviral signaling has been shown to suppress global host translation to fight geminiviruses and tobacco rattle virus (TRV) [16,17,18]. This minireview focuses on the three layers of receptor-mediated plant antiviral immunity (PTI, ETI, and NIK1-mediated antiviral signaling) and describes the virulence strategies evolved by geminiviruses to overcome these host defense barriers. We also discuss the interplay of antibacterial PTI with the NIK1-mediated antiviral signaling, which may circumvent the NIK1 inhibition by the geminivirus NSP.

## 2. Genome Organization of Geminiviruses (*Geminiviridae* Family)

**Figure 1:**
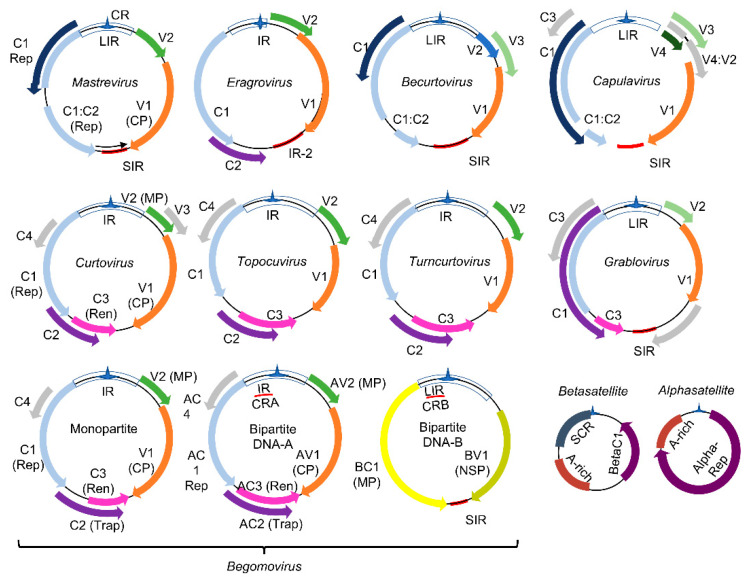
Genomic organization of geminiviruses (*Geminiviridae* family): The *Geminiviridae* family includes nine genera represented by monopartite or bipartite species. LIR denotes the long intergenic region, SIR, the short intergenic region, and CR, the common region. The viral proteins replication initiator protein, Rep (C1), and replication enhancer protein, Ren (C3), are associated with replication, and the transcription activator protein, Trap (C2), with the transcription of viral and host genes; AC4 is a virulence factor. The capsid protein (CP) is indicated in the monopartite and bipartite genomes. In monopartite species, V2 represents the movement protein (MP). In bipartite begomoviruses, MP (BC1) is encoded by the DNA-B that also encodes the nuclear shuttle protein, NSP (BV1), which facilitates the nucleocytoplasmic movement of viral DNA. Bipartite begomoviruses are often associated with DNA satellites: the alphasatellites, which encode a replication protein (Rep), and the betasatellites that encode the virulence-related βC1 protein. A-rich is a conserved adenine rich region of the DNA satellites, and SCR is the satellite conserved region. As with βC1, the encoded products of ORFs V1, V2, BV1, C2, C4, and AC3 from some geminivirus species are also reported as virulence factors. Adapted from [19].

The *Geminiviridae* family represents a group of plant DNA viruses that cause severe diseases in many crops, a major constraint to agricultural productivity and food security. This family includes nine genera (*Mastrevirus*, *Eragrovirus, Becurtovirus*, *Capulavirus*, *Curtovirus*, *Topocuvirus*, *Turncurtovirus*, *Grablovirus*, and *Begomovirus*), which comprise viruses with monopartite or bipartite circular single-stranded DNA genomes of 2.6 to 3.0 kb [19] (Figure 1). The viral genomes replicate via dsDNA intermediates containing complementary-sense (CS) and virion-sense (VS) strands (Figure 1). Proteins encoded by the CS strand are called C1-C5 and by the VS strand, V1 and V2. In bipartite begomoviruses, the ORF designations incorporate A (AC1−AC5, AV1−AV2) or B (BC1, BV1) from DNA-A and DNA-B. These designations are often replaced with protein function, including replication initiator protein (Rep/AC1/C1), transcriptional activator protein (TrAP/AC2/C2), replication enhancer protein (REn/AC3/C3), coat protein (CP), movement protein (MP/BC1), and nuclear shuttle protein (NSP/BV1). Due to the limited coding capacity of the viral genomes, the encoded proteins evolved into multifunctional proteins to harbor both the virus cycle-supporting and host immunity-suppressing functions. 

## 3. Geminiviruses Activate and Suppress Viral PTI and ETI-Like Responses

Although viruses are intracellular parasites, recent studies have demonstrated that virus infection can activate PTI-like responses in the host [7,20]. However, viral PAMPs and their cognate PRRs have not been isolated and characterized, and hence, the mechanism of PTI activation in response to viral infection remains unknown. Fragmented knowledge about viral PTI includes the observation that RNA from infected plants and double-stranded (ds) RNA [poly(I:C)] have been shown to trigger typical PTI responses in a somatic embryogenesis receptor kinase 1 (SERK1)-dependent manner [21,22]. SERK1 belongs to the subfamily II of the transmembrane leucine-rich repeat (LRR) receptor-like kinases (RLK) that encompasses several characterized coreceptors of PRRs [6,23]. Viral dsRNA may be regarded as potential PAMPs as it is usually generated in infected plants by RNA and DNA viruses [7,21]. However, PRRs recognize nonviral PAMPs extracellularly, and as intracellular obligate parasites, it is uncertain whether plant PRRs would perceive viruses via its extracellular PAMP-sensing domain. 

Resembling the dsRNA-mediated SERK1 activation, NIK1, another LRR-RLK member of the subfamily II of coreceptors, can be activated by begomovirus-derived nucleic acids [7]. However, NIK1 activation induced by exogenously provided viral PAMPs requires mechanically injured leaves to facilitate the entry of begomovirus-derived nucleic acids in noninfected *Arabidopsis thaliana* leaf cells. These results suggest that PRRs sense viral PAMPs intracellularly, which would require either an endocytic internalization of the PRR sensing extracellular domain or perception via the PRR kinase cytosolic domain. In mammalian cells, the perception of virus-derived nucleic acids (DNA, ssRNA, and dsRNA) occurs intracellularly by the LRR domain of endosomal toll-like receptors (TLRs) or by the kinase sensor domain of cytosolic receptors [24,25]. An example of mammalian dsRNA-sensing kinase domain includes the protein kinase RNA-activated (PKR), which phosphorylates eIF2 alpha to shutdown translation in response to virus infection [25]. The recent demonstration that dsRNAs induce phosphorylation of eIF2 alpha in Arabidopsis may suggest a similar mechanism for viral PAMP-mediated kinase sensor domain activation in plant cells [26].

**Figure 2:**
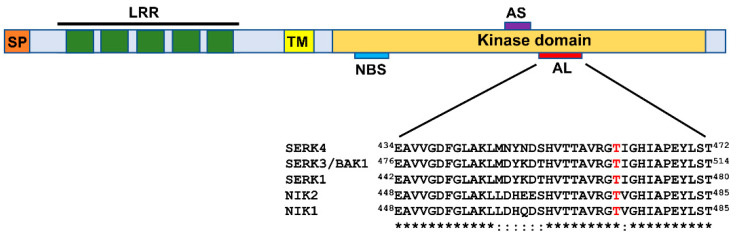
Domain organization of the LRR-RLK subfamily II members: Leucine-rich repeat receptor-like kinases (LRR-RLKs) belonging to the subfamily II harbor a signal peptide (SP) and an LRR domain with 5 repeats at the N-terminal extracellular region, a transmembrane segment (TM), and a kinase domain at the cytosolic side. The relative positions of the nucleotide-binding site (NBS), the active site (AS), and the activation loop (AL) of the kinase are indicated. In the A-loop sequence comparison of LRR-RLK subfamily II representatives, the phosphorylation-dependent activation site of the kinase is highlighted in red.

In addition to SERK1, required for viral PTI activation, the subfamily II of LRR-RLKs comprises members that function as coreceptors for multiple complexes of RLKs involved in defense [6,23]. The receptor configuration of the members of the LRR-RLK subfamily II includes an N-terminal extracellular domain harboring four complete LRRs and a fifth incomplete LRR, a single-pass transmembrane segment, and a conserved serine/threonine kinase domain at the cytosolic side [27,28] (Figure 2). Among members of the LRRII-RLK clade, brassinosteroid insensitive 1 (BRI1)-associated kinase 1 (BAK1), also designated SERK3, represents the best-characterized coreceptor that forms complexes with multiple PRRs, assembling active immune complexes to initiate nonviral and viral PTI [6,29]. Examples of PRRs for BAK1 coreceptor include the flagellin receptor flagellin sensing 2 (FLS2), elongation factor-thermo unstable (EF-Tu) receptor (EFR), or PEP1 receptor 1 (PEPR1), which perceive specific PAMPs/DAMPs and trigger PTI (reviewed in [6]). Additional LRRII-RLK subfamily members, SERK4/BKK1 (BAK1-like kinase 1) and NIK1, have also been shown to function in antiviral immunity [30,31]. 

**Figure 3:**
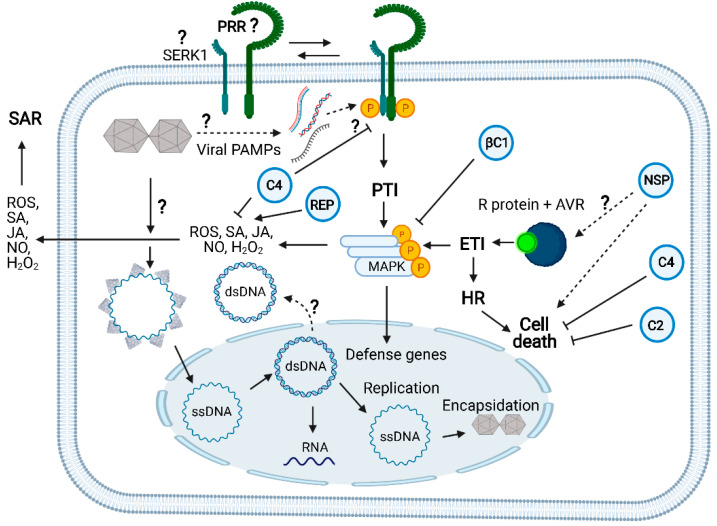
Receptor-mediated innate immunity against geminiviruses and counterdefensive viral activities: Like any other plant virus, geminiviruses are obligate intracellular parasites delivered into the cytoplasm of plant cells by the insect vector. The viral particle unpacking is likely to occur in the cytoplasm. Then, viral (v) CP-bound ssDNA is directed to the nucleus where v-ssDNA is converted to v-dsDNA to replicate the viral genome via the rolling circle mechanism and for transcribing the viral genes. The geminivirus infection both induces and suppresses the plant innate immunity. Geminivirus-derived nucleic acids may act as a viral PAMP to induce the dimerization of a yet-to-be-identified PRR with its coreceptor SERK1 to initiate the PTI signaling pathway. SERK1 has been shown to be required for viral PTI elicited by dsRNA, but no direct evidence exists for its participation in geminiviral PTI. FLS2 may function as a geminiviral PRR because C4 acts as a virulence effector by binding to FLS2 and inhibiting early PTI responses. Likewise, the betasatellite βC1 virulence effector affects the MAPKinase cascade. In a resistant *Phaseolus vulgaris* genotype to bean dwarf mosaic virus (BDMV), NSP acts as an avirulence factor; it is recognized by an unknown intracellular receptor and activates HR, cell death, and ETI-like responses. A typical NLR intracellular receptor of ETI (TYNBS1) confers resistance to TYLCV, yet the cognate geminivirus avirulence factor is unknown. Rep has been shown to induce ROS and SA-mediated defenses. As virulence effectors, C4 and C2 counter ETI activation by suppressing HR and cell death. C4 also inhibits SA-mediated defenses. The question marks indicate either events not well clarified or unknown. Solid line arrows describe experimentally demonstrated events and dotted line arrows denote genetically implicated events. See Figure 1 for the designations of the viral proteins. The figure was created with BioRender 101 (BioRender.com; https://biorender.com/, accessed on 1 April 2021).

Although nucleic acid-sensing PRRs have yet to be identified in plants, virus infection induces several PTI marker immune events, indicating that viral PTI may operate in plants with similar mechanisms as nonviral PTI [7,21,32]. Furthermore, PTI preactivation with nonviral PAMPs increases resistance against viruses, and PTI inhibitors enhance susceptibility to virus infection [33,34]. Reverse genetics studies targeting upstream components of PTI have also confirmed that plants employ PTI to fight virus infection. Inactivation of the PTI coreceptors BAK1, SERK1, and SERK4/BKK1 has been shown to enhance susceptibility to RNA virus infection [8,22,29,31]. Precedents in the literature indicate that geminivirus infection activates and suppresses PTI (Figure 3). Rep from different geminiviruses induces PTI-associated marker genes and SA-dependent defenses [35,36]. When coexpressed with tomato yellow leaf curl virus (TYLCV) C4 protein, Rep redirects C4 to the chloroplasts where it acts as a PTI suppressor by reducing SA and ROS-dependent defense signals [20]. Geminivirus infection promotes the plasma membrane-bound N-myristoylated C4 translocation to the chloroplasts, where the nonmyristoylated C4 interacts with the plant calcium-sensing receptor (CAS) and hampers SA biosynthesis and mediated defenses [37]. In addition to Rep and C4, tomato yellow leaf curl China virus (TYLCCV) betasatellite βC1 protein, required for symptom induction, has been shown to interfere with PTI-like responses. βC1 affects PTI-induced MAPK activation and downstream responses by targeting MKK2 in *A. thaliana* and *Nicotiana benthamiana* [38,39]. 

TYLCV C4 also interacts with RLKs, including FLS2, a typical PRR activated by the bacterial PAMP flagellin, and NIK1, an antiviral immune receptor that can protect plants against begomovirus [3,40]. The C4 interaction with FLS2 has been shown to inhibit partially PTI-like responses [40]. The C4 ectopic expression in Arabidopsis inhibited the early apoplastic ROS burst following flg22 perception but not the expression of PTI-marker defense genes, and neither affected the late inhibition of growth mediated by PTI activation. Likewise, NSP has been shown to interact with the FLS2 coreceptor BAK1 and hence may impair immune responses upon pathogen perception, similarly to the NIK1 inhibition by the begomovirus-encoded NSP [23,41]. However, NSP-BAK1 interaction has only been shown by the yeast two-hybrid system; therefore, further studies are necessary to assign a PTI-suppressing function to NSP. 

The second layer of plant innate immunity, ETI, represents a more specific and robust line of host defense, which, in contrast to PTI, often results in programmed cell death, the hypersensitive response (HR), to restrict the pathogen to the site of infection [42]. ETI relies on intracellular immune receptors (resistance, R, proteins), which recognize, directly or indirectly, very specifically pathogen avirulent effectors to activate immune responses (11,14,20]. The antiviral intracellular R proteins are mostly represented by the nucleotide-binding leucine-rich repeat (NLR) receptors [14,43,44]. The natural Ty-2 resistance locus to TYLCV encodes an NB-LRR protein, named TYNBS1, which might activate ETI via recognition of a yet-to-be-identified geminiviral effector [45]. Examples of non-NB-LRR resistance proteins against TYLCV include the sensor proteins Ty-1 and Ty-3, which encode an RNA-dependent RNA polymerase (RDR) [46] and the recessive resistance gene encoded by the Ty-5 locus, the messenger RNA surveillance factor Pelota [47,48]. However, Ty-1-based resistance against TYLCV is uncoupled to ETI and involves enhanced transcriptional gene silencing [49], and the underlying mechanism mediating Pelota resistance against geminiviruses is unknown.

Further evidence that geminiviruses activate ETI is derived from studies of geminivirus inducers and suppressors of HR and ETI-like responses (Figure 3). Geminivirus infection induces hypersensitive response (HR) and senescence-related genes without developing a visible cell death phenotype [50]. More specifically, the geminivirus proteins Rep, NSP, and V2 have been shown to induce HR [51,52,53]. In contrast, some geminiviral proteins possess the capacity of suppressing HR-like cell death [19]. The C2 protein from papaya leaf curl virus (PaLCuV) and cotton leaf curl Kokhran virus (CLCuKoV) has been shown to inhibit V2-mediated HR [53]. Likewise, TYLCV infection alleviates cell death in tomato plants, induced by the inactivation of heat shock protein 90 (HSP90) and suppressor of the G2 allele of Skp1 (SGT1) [54]. Still, the TYLCV-mediated cell death suppression mechanism is unknown. In contrast, the underlying mechanism for the cell death-suppressing activity of C4 from tomato leaf curl Yunnan virus (TLCYnV) has been recently uncovered [55]. C4 interacts with HIR1, impairs HIR1 self-oligomerization, and promotes its degradation, thereby inhibiting the HIR1-mediated HR and increasing virus pathogenicity. Although several lines of evidence indicate that both monopartite and bipartite begomoviruses induce and suppress HR, the mechanisms of ETI activation and suppression by geminiviruses are still far from understood.

## 4. NIK1-Mediated Antiviral Signaling and Crosstalk with Antibacterial Immunity

NIK1 was first identified in tomato as a virulence target of NSP from tomato golden mosaic virus (TGMV). It transduces an antiviral signal that culminates in the suppression of global host translation [16,18]. NIK1 interaction with NSP is conserved among NIKs from different hosts, such as Arabidopsis, soybean, and tomato, and NSPs from distinct begomoviruses, including cabbage leaf curl virus (CabLCV), TGMV, tomato crinkle yellow leaf virus (TCrYLV), and tomato yellow spot virus (ToYSV) [30,56]. Recently, NIK2 from Arabidopsis has been shown to function as a NIK1 paralog in the antiviral signaling pathway [7,27].

Although the NIK1-mediated antiviral signaling has been considered a new paradigm for plant antiviral immunity, it exhibits several PTI features [57]. First, NIK1 is structurally related to the typical PTI coreceptors BAK1, SERK4, and SERK1, which display a highly conserved kinase domain and a similar configuration of the LRR extracellular domain [27] (Figure 2). Second, NIK1 activation requires phosphorylation at threonine-474, a conserved position among the activation sites of BAK1, SERK4, and SERK1, which may indicate a similar mechanism for kinase activation [58]. Third, NIK1 is activated by begomovirus-derived nucleic acids, resembling the PTI activation mechanism by viral PAMPs [7,22]. Finally, the NIK1-mediated antiviral signaling is inhibited by begomovirus-encoded NSP, fulfilling the premise that the successful ETI activation would depend on the PTI suppression by viral effectors [7,11]. Examples of PTI suppressors from other plant viruses include CP from plum pox virus (PPV), *Potyvirus* genus [8], P6 from cauliflower mosaic virus (CaMV), *Caulimovirus* genus [59], and MP from cucumber mosaic virus (CMV), *Cucumovirus* genus [9]. Therefore, like the classic viral PTI, the NIK1-mediated antiviral signaling is activated and repressed by begomovirus infection. 

**Figure 4:**
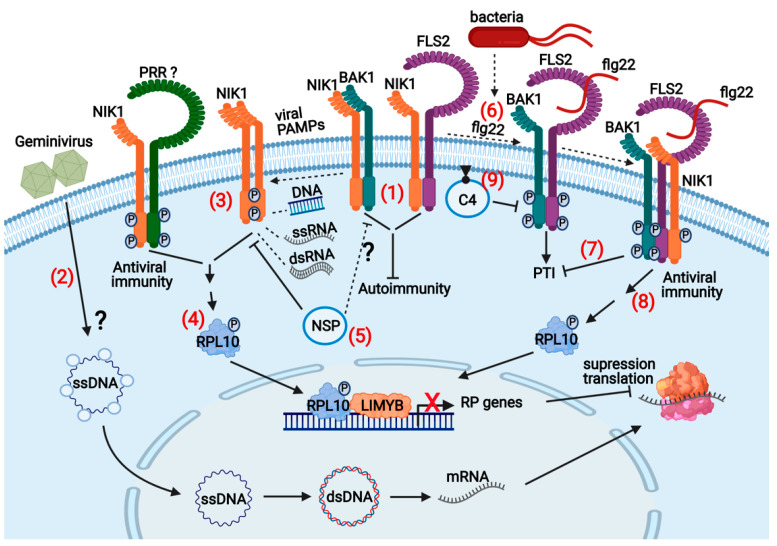
NIK1-mediated antiviral signaling and crosstalk with antibacterial immunity: In noninfected cells, (1) NIK1 pools are bound to the PRR FLS2 and the PTI coreceptor BAK1 to prevent autoimmunity. (2) At the onset of infection, CP-bound ssDNA is transported to the nucleus for replication of the viral genome and transcription of viral genes. (3) In the infected cells, geminivirus-derived nucleic acids act as viral PAMPs and induce the dimerization of NIK1 with itself or with another unknown receptor (PRR-like) for transphosphorylation of the kinase intracellular domains. NIK1 phosphorylation at Thr-474 activates the kinase that in turn mediates the RPL10 phosphorylation. (4) Phosphorylated RPL10 is redirected to the nucleus, where it interacts with LIMYB to repress the expression of ribosomal proteins (RBs) and translational machinery-related genes, culminating in suppressing host and viral mRNA translation. (5) NSP counters this antiviral mechanism’s activation by binding to the NIK1 kinase domain, thereby preventing NIK1 phosphorylation and activation. (6) Bacterial infection (*Pseudomonas* spp) provides the apoplastic bacterial PAMP flagellin (flg22), which is recognized by PRR FLS2, thereby inducing the formation of the active immune complex FLS2-BAK1 to initiate PTI. FLS2-BAK1-bound NIK1 is then phosphorylated at Thr-474 by the activated BAK1, (7) enhancing NIK1 suppression of PTI but (8) activating the NIK1-mediated antiviral signaling that results in suppression of global translation. (9) C4 from geminiviruses binds to FLS2 and inhibits the early PTI responses and subsequent NIK1 activation creating an environment that favors virus infection. The question marks indicate either events not well clarified or unknown. The figure was created with BioRender 101.

Despite the similarities between PTI and NIK1 signaling, the downstream events of receptor/coreceptor activation and the assembly of a defense response differ considerably. Typically, the PTI activation results in ROS accumulation as an early response, followed by activation of MAP Kinases, induction of PTI-associated defense genes, ethylene and salicylic acid synthesis, and callose deposition [5]. Differing from PTI immune responses, NIK1-mediate antiviral signaling results in suppression of host global translation (Figure 4) [60]. According to the mechanistic model for NIK1 activation, during infection, begomovirus-derived nucleic acids function as viral PAMPs that induce NIK1 dimerization with itself, NIK2, or an unknown receptor (Figure 4) [7,30]. As members of the subfamily II of LRRII-RLK with similar ectodomain configuration as PTI coreceptors, Arabidopsis NIK1 and its paralog NIK2 may function as coreceptors of unknown nucleic acid-sensing PRRs [6]. An active immune complex formation leads to NIK1 phosphorylation at Thr-474, essential for NIK1 kinase activation to initiate signaling [41,58]. Activated NIK1 mediates the phosphorylation of the downstream component ribosomal protein (RP) L10, which in turn promotes its translocation to the nucleus [61,62]. NIK1 may control the extent of RPL10 phosphorylation by sequential autophosphorylation at the functionally antagonistic Thr-469 residue [41]. In the nucleus, phosphorylated RPL10 interacts with the transcriptional repressor L10-interacting Myb-domain-containing protein (LIMYB) to fully repress the expression of translational machinery-related genes, causing suppression of global protein synthesis [18]. Viral mRNAs cannot escape the host translational suppression; they are not translated efficiently, compromising the infection [16,18]. Counteracting the activation mechanism of the defense pathway, NSP interacts with the kinase domain to prevent NIK1 phosphorylation and activation, creating an environment that is more favorable to begomovirus infection [30,41,61]. Therefore, the NIK1-mediated antiviral signaling is an evolutionarily suppressed host defense by begomoviruses.

The NIK1-mediated immunity cross-communicates with PTI because NIK1 and NIK2 also function as PTI suppressors (Figure 4) [17]. Inactivation of either NIK1 or NIK2 in Arabidopsis enhances PTI activation and confers resistance to *Pseudomonas syringae*, an opposite phenotype displayed by NIK-overexpressing lines. Under normal conditions, NIK1 interacts with the flagellin receptor FLS2 and its coreceptor BAK1 to interfere with the immune complex formation and prevent autoimmunity (Figure 4). The bacterial PAMP flagellin, or its active peptide flg22, interacts with FLS2 and induces dimerization with BAK1, leading to the formation of an active immune complex to initiate PTI signaling. FLS2-mediated activation of BAK1 promotes NIK1 phosphorylation at the activation site Thr-474, which stimulates further the NIK1 interaction with FLS2 and BAK1 to control the extent of PTI activation and in turn triggers the activation of the NIK1-mediated antiviral signaling. Flg22-induced NIK1 phosphorylation requires both PRR FLS2 and its coreceptor BAK1, indicating that NIK1 acts downstream of receptor signaling. These results substantiate the argument that NIK1 may function as a coreceptor in ligand-induced activation of a yet unknown nucleic acid-sensing receptor (PRR) to elicit the NIK1-mediated antiviral defense. They also suggest that a bacterial PAMP can activate begomovirus resistance via NIK1 phosphorylation, which may alleviate NSP inhibition. Several lines of evidence indicate that the phosphorylation-mediated activation of NIK1 is downstream of NIK1 inhibition by begomovirus-encoded NSP. First, the NSP-binding site on NIK1 maps within an 80 amino acid stretch overlapping the activation loop and the kinase activation site (Thr474) [30]. Second, the replacement of the Thr-474 activation site with an aspartate creates a phosphomimic mutant that is no longer inhibited by NSP [41]. Finally, overexpression of the phosphomimic mutant NIK1-T474D in Arabidopsis and tomato plants confers broad-range resistance to begomovirus infection [16,18]. Therefore, treating hosts with the bacterial PAMP flg22 prior to begomovirus infection may lead to an efficient NIK1-mediated antiviral immune response by bypassing NSP inhibition. This interplay between immune responses allows bacteria to activate immunity against viruses, hence preventing multiple infections by pathogens from different kingdoms presumably to avoid competition. 

## 5. Conclusions

Recent studies have demonstrated that plants deploy both levels of the classic innate immunity, PTI and ETI, to fight virus infection, and geminiviruses are not exception. Nevertheless, evidence for geminiviral PTI and ETI relies on the observations that geminivirus infection can induce and suppress PTI and ETI-like responses. In addition, some geminiviral proteins function as PTI suppressors, and at least one isolated resistance gene to TYLCV displays the classical NL-LRR configuration of intracellular R proteins that elicits ETI. However, our knowledge about geminiviral PTI and ETI mechanisms is still rudimentary, and several steps in PTI and ETI activation and response are unknown. For instance, we do not know the identity of geminiviral PAMPs that would induce PTI, and the cognate PRRs have not been identified. Furthermore, the repertoire of PTI geminiviral suppressors is restricted to C4 and perhaps to βC1 and NSP. The NSP-suppressed NIK1-mediated antiviral signaling has been shown to cross-communicate with bacterial PTI. In addition to suppressing global host translation, NIK1 activation leads to suppression of bacterial PTI, which restricts even further the use of sustained NIK1-mediated antiviral signaling to control virus infection. The isolation of geminiviral PAMPs and cognate PRRs, geminiviral effectors (Avr genes), and NL-LRR host targets will help to elucidate the mechanism of ETI activation and PTI suppression as the foundation for understanding the evolutionary pressure dynamics acting upon geminivirus and host interactions. This understanding will ultimately help to determine how to deploy the immune system to control geminivirus infection.
